# A multilevel analysis of prenatal care and birth weight in Kenya

**DOI:** 10.1186/s13561-014-0033-3

**Published:** 2014-11-23

**Authors:** Japheth Osotsi Awiti

**Affiliations:** School of Economics, University of Nairobi, University Way, Nairobi, Kenya

**Keywords:** Birth weight, Prenatal care, Multilevel analysis, Kenya

## Abstract

The paper investigates the effect of adequate use of prenatal care on birth weight in Kenya using data from the Kenya Demographic and Health Survey of 2008–2009 together with additional administrative data. Both a single–level model and a multi–level model are estimated. The estimation strategy controls for potential sample selection bias, potential endogeneity of prenatal care, and potential unobserved heterogeneity. The results indicate that adequate use of prenatal care increases birth weight, holding other factors constant. We further observe that the single–level model overstates the effect of prenatal care on birth weight. The results imply that infant health can be improved by using prenatal care adequately. The study calls for the pursuit of policies that encourage adequate use of prenatal care by expectant mothers such as ensuring availability of skilled health care providers such as doctors and nurses at prenatal care clinics, reducing the average distances mothers have to cover when seeking prenatal care services, intensifying education of females as a way of empowering them to be able to make the right choices regarding when to seek prenatal care and from whom, and increasing income opportunities for households.

## Background

The study of infant health^a^ is important because many health problems that we observe in adult life originate in the early years of life [1]. Infant health can be measured at both the individual and population levels. Examples of indicators of infant health at the population level include neonatal mortality rate, post–neonatal mortality rate, infant mortality rate, birth weight distribution, and gestational age distribution [2,3]. Examples of infant health indicators at the individual level include child survival, birth weight, Apgar score, gestation, disability, and nutritional indicators [4,5].

Table 1 gives data on some key infant health indicators for selected Sub–Saharan African countries and other regions.

**Table 1:** **Some key infant health indicators for selected Sub-Saharan Africa countries**

**Country/region**	**Neonatal mortality rate**			**Infant mortality rate**			**Low birth weight**
	**(per 1,000 live births)**			**(per 1,000 live births)**			**newborns (%)**
	**2000**	**2009**	**2012**	**2000**	**2009**	**2012**	2000–2009*
Angola	48	42	45	126	98	100	12
Botswana	32	22	29	66	43	41	13
Cameroon	37	37	28	96	95	61	11
Central African Republic	47	45	41	119	112	91	13
Democratic Republic of Congo	51	51	44	126	126	100	10
Eritrea	23	17	18	58	39	37	14
Ethiopia	43	35	29	91	67	47	20
Gabon	28	25	25	61	52	42	14
Gambia	37	32	28	93	78	49	20
Ghana	35	26	28	68	47	49	13
Kenya**	**32**	**27**	**27**	**66**	**55**	**49**	**8**
Lesotho	42	33	45	86	61	74	13
Liberia	51	37	27	133	80	56	14
Madagascar	31	21	22	65	40	41	16
Malawi	37	30	24	99	69	46	14
Mauritius	12	9	9	16	13	13	14
Mozambique	47	41	30	123	96	63	15
Namibia	26	19	18	50	34	28	16
Nigeria	46	39	39	114	86	78	12
Senegal	36	31	24	61	51	45	19
Sierra Leone	56	49	50	150	123	117	14
Swaziland	26	20	30	71	52	56	9
Togo	36	32	33	78	64	62	12
Uganda	34	31	23	94	79	45	14
United Republic of Tanzania	39	34	21	86	68	38	10
Zambia	35	29	29	99	86	56	11
Zimbabwe	27	34	39	69	56	56	11
**WHO Region**							
African	41	36	32	98	80	63	13
Americas	13	9	8	22	15	13	8
South – East Asia	39	31	27	62	45	39	24
European	10	7	6	19	12	10	7
Eastern Mediterranean	35	30	26	65	54	44	21
Western Pacific	17	11	9	28	18	14	5

^*^Data is for the latest year available.

^**^The boldface is to help the reader to quickly locate the Kenyan data in the table.

Source: [6,7].

A closer look at the data shows that most of the Sub-Saharan African countries have poor infant health outcomes. For example, Kenya had a neonatal mortality rate of 27 per 1,000 live births in 2012 while Tanzania had an infant mortality rate of 38 per 1,000 live births in 2012. Further, 8% of the infants in Kenya and 10% of the infants born in Tanzania have low–birth weight. The table also shows that about 13% of the infants born in the African region have low–birth weight as compared to only 5% in Western Pacific, 7% in Europe and 8% in the Americas. A higher percentage of infants with low–birth weight are, however, found in South–East Asia and the Eastern Mediterranean region as compared to Africa.

Additional indicators of infant and child health for Kenya show that 85 of every 1,000 infants born alive in Kenya in 2010 were likely to die before reaching their fifth birthday, 15% of all under–five deaths in Kenya in 2010 were due to premature births, and about 16% of children under 5 years of age in Kenya are underweight [8].

There is, therefore, need to find out how infant health can be improved in Kenya. This study investigates one way in which this can be done.

Although there are many indicators of infant health, this study focuses on birth weight. Since birth weight represents the outcome of the gestation period, it is a good measure of infant health at birth [3]. Weight at birth of less than 2,500 grams is termed to be low [9]. Low birth weight is associated with various adverse health outcomes such as fetal and neonatal morbidity and mortality, impaired cognitive development, and the advent of chronic diseases in later life [10,11].

The literature on the determinants of low birth weight is expansive (see, for example, [10-13]). The literature identifies a number of maternal risk factors^b^ for low birth weight. The factors include historical factors (such as short or long birth interval), demographic factors (such as adolescent mothers), nutritional factors (such as iron deficiency), anthropometric factors (such as low body mass index), medical and pregnancy–related conditions (such as malaria infection), adverse psychosocial factors, lifestyle–related factors (such as tobacco use), environmental tobacco exposure, violence/maternal abuse, infertility and in vitro fertilization (IVF) treatment, and health care risks (such as inadequate prenatal care) [10-12].

Prenatal care, also called antenatal care, refers to the health care provided to an expectant mother throughout the period of pregnancy [14,15]. In the ideal scenario, prenatal care should involve the following activities: provision of appropriate advice on health matters such as nutrition, hygiene, newborn care and safer sex; identification of expectant women at risk of experiencing pregnancy complications through appropriate screening and diagnosis; and either the treatment of identified pre–existing illnesses and conditions or, where treatment is not available at the particular health facility, referral to an appropriate health facility that can deal with the identified conditions [14]. Prenatal care can benefit both expectant mothers and their unborn children through identification of expectant mothers at risk of delivering infants with low–birth weight or experiencing complications during delivery and providing appropriate psychosocial, nutritional, and medical interventions aimed at reducing such risks [11,16].

Several indicators have been used in the literature to measure prenatal care use. Examples of these indicators include number of prenatal care visits, number of prenatal care visits adjusted for pregnancy length, whether prenatal care was ever initiated, author–constructed quality index of type of care received, timing of first prenatal care visit, Kessner index of adequacy of prenatal care received, adequacy of prenatal care utilization index, and indexes based on World Health Organization (WHO) recommendations for developing countries [17-19].

The World Health Organization (WHO) recommends a minimum of four prenatal care visits at particular intervals, to skilled health personnel (doctors or nurses), for expectant women in developing countries [14]. There is also a recommended timing for each visit. For example, it is recommended that the first prenatal care visit should be made within the first 16 weeks of pregnancy while the third visit should be made at 32 weeks of pregnancy [14]. There are further detailed recommendations on what should be done at each visit [14]. It has been shown that the recommendations of WHO regarding prenatal care use in developing countries are appropriate [15]. In this study, we construct a prenatal care utilization index based on WHO’s recommendations.

A careful look at the literature reveals that there is still controversy over the effectiveness of prenatal care in improving birth weight. Although there are studies which show that prenatal care improves birth weight (see, for example, [3,10,17,19-21]), there are still others that find prenatal care to be ineffective in improving birth weight (see, for example, studies cited in [11]). Yet other studies (see, for example, [22]) only find weak influences of prenatal care on the health of infants. A look at the literature further reveals that there are very few studies in Sub–Saharan Africa investigating the effect of prenatal care on birth weight. Most of the studies cited in the literature also use a single–level model intheir analysis.

This study investigates the effect of adequate use of prenatal care on birth weight in Kenya. The main objective of the study is to, therefore, show how adequate use of prenatal care affects birth weight in Kenya, controlling for the effects of other potential determinants of birth weight.

Specifically, in the study, we first construct a measure of adequacy of prenatal care use in Kenya following the WHO recommendations. Second, we determine the factors influencing adequate utilization of prenatal care in Kenya. Third, we establish the effect of adequate use of prenatal care on birth weight in Kenya, using both single–level and multi–level analysis. Fourth, by comparing the results of the single–level model and the multi–level model, we attempt to make a theoretical contribution by determining the most appropriate way of modelling the effect of prenatal care on birth weight. Finally, we draw appropriate policy implications from the study findings.

The study contributes to the literature by adding to the studies that find prenatal care to be effective in improving birth weight. It also contributes to the literature by studying a Sub–Saharan African country, Kenya. Finally, unlike previous studies, our study estimates both a single–level model and a multi–level model that links prenatal care use to birth weight and demonstrates that the effects of prenatal care on birth weight are overstated in the single–level model.

## Methods

In this section, we present the theoretical framework, the conceptual model used in the analysis, the identification strategy, the empirical model, and a discussion of the data used in the analysis.

### Theoretical framework

Following [3,23,24], we assume that an expectant mother, *j*, maximizes the utility, *U*_*j*_, obtained from her consumption of various goods and services that have no impact on the health of her unborn child, *X*_*j*_, and the health status of her unborn child, *H*_*j*_. We can represent the expectant mother’s utility function as follows:

(1) $$ U_{j}=U(X_{j}, H_{j}).  $$

We assume that the health status of the unborn child, *H*_*j*_, is in turn influenced by the adequacy of prenatal care use, *Z*_*j*_, that affects health directly, other factors, *Y*_*j*_, and unobservable biological endowments, *μ*_*j*_. The health production function of the unborn child can, therefore, be represented by the following:

(2) $$ H_{j}=H(Z_{j}, Y_{j}, \mu_{j}).  $$

The mother is assumed to maximize her utility function subject to the above health production function and a budget constraint given by:

(3) $$ I_{j}=P_{x}X_{j}+P_{y}Y+P_{z}Z_{j}  $$

where *I* is exogenous mother’s/household’s income, *P*_*x*_ is the unit price of *X*, *P*_*y*_ the unit price of *Y*, and *P*_*z*_ is the unit price of *Z*.

Following [3], we can manipulate the above equations to obtain the input demand equations shown below,

(4) $$\begin{array}{*{20}l} X_{j}&=&X(P_{x}, P_{y}, P_{z}, I_{j}, \mu_{j}) \end{array} $$

(5) $$\begin{array}{*{20}l} Y_{j}&=&Y(P_{x}, P_{y}, P_{z}, I_{j}, \mu_{j}) \end{array} $$

(6) $$\begin{array}{*{20}l} Z_{j}&=&Z(P_{x}, P_{y}, P_{z}, I_{j}, \mu_{j}) \end{array} $$

We can derive the effects of the changes in the prices of the various goods and services on infant health as follows [3]:

(7) $$\begin{array}{*{20}l} \frac{dH}{{dP}_{x}}&=&H_{z}\frac{dZ}{{dP}_{x}}+H_{y}\frac{dY}{{dP}_{x}} \end{array} $$

(8) $$\begin{array}{*{20}l} \frac{dH}{{dP}_{y}}&=&H_{z}\frac{dZ}{{dP}_{y}}+H_{y}\frac{dY}{{dP}_{y}} \end{array} $$

(9) $$\begin{array}{*{20}l} \frac{dH}{{dP}_{z}}&=&H_{z}\frac{dZ}{{dP}_{z}}+H_{y}\frac{dY}{{dP}_{z}} \end{array} $$

where *H*_*z*_ is the marginal product of the health input *Z* and *H*_*y*_ is the marginal product of the health input *Y*.

The above equations demonstrate that input prices are correlated with infant health [3]. This is mainly because the changes in input prices result in changes in the quantities of inputs used in the production of health. The changes in the quantities of health inputs, in turn, lead to changes in the health status of the infant. There is, therefore, an indirect effect of input prices on infant health. The consequences for the policy makers here is that sometimes health can be changed in the desired direction by pursuing policies that change the prices in the appropriate direction.

### Conceptual model

**Figure 1:**
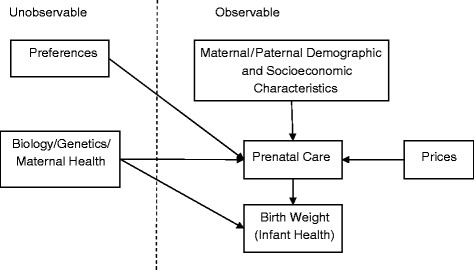
**Conceptual model for analyzing the effect of prenatal care on birth weight.** This figure shows the conceptual model that is used to analyze the effect of prenatal care on birth weight.

We can develop the conceptual model shown in Figure 1 for the analysis of the effect of prenatal care use on birth weight following [4].

According to the figure, birth weight (a measure of infant health) is influenced by prenatal care use and unobservable biological endowments of both the mother and the child, including true maternal health status.

Prenatal care use, in turn, is influenced by maternal/ household demographic and socio–economic characteristics, community characteristics or environmental factors, prices, and unobservable maternal/household preferences.

### Estimation issues

Our objective is to consistently estimate Equation (2) so that we can be able to tell the effects of changes in *Z* (prenatal care) on *H* (health status of the infant). Such estimation is straightforward in the absence of challenges. Depending on how *H* is measured and on the specific functional form, all we need to do is find the necessary data and then use the appropriate estimation technique.

Sometimes, however, there are challenges such as the values of *H* missing in the dataset for some of the observations, correlation between the error term in the model and *Z*, and non–linear interaction between *Z* and some unobservable factors that causes the effect of *Z* on *H* to differ amongst population subjects [25-27]. These challenges pose difficulties to the estimation process and have to be addressed if we are to get consistent estimates. The challenges may call for use of a different estimation technique or the modification of the model to be estimated before the estimation can be done.

The challenge of missing values of *H* for some of the observations leads to a problem of potential sample selection bias, the challenge of correlation between the error term and *Z* leads to a problem of potential endogeneity in the model, and the challenge of non–linear interaction between *Z* and unobservable factors that cause differences in the effect of *Z* on *H* amongst population subjects leads to the problem of potential unobserved heterogeneity [25-27].

#### Sample selection bias

In general, sample selection bias is likely to occur in situations where the dependent variable is observed only for a restricted, non–random sample [25]. It is likely to arise when we examine a subsample in circumstances where the unobservable factors that influence inclusion of individuals in the subsample are correlated with the unobservable factors that influence the variable of primary interest [28]. For example, in our case, we only observe the birth weight of a child if it is reported in the dataset. The birth weight information is, however, missing for about 52% of the children.

In this case, sample selection bias will occur if the unobservable factors affecting the decision to report the birth weight of the child are correlated with the unobservable factors affecting the birth weight itself.

Although several approaches to correcting for sample selection bias have been proposed in the literature (see, for example, [29,30]), we use the approach suggested by Olsen [30]^c^. Unlike the popular Heckman approach [29] which is based on maximum likelihood estimation^d^, the Olsen approach only requires Ordinary Least Squares (OLS) regression techniques in the first step [30].

#### Endogeneity

In our model, we suspect that the covariate measuring the adequacy of prenatal care use is endogenous^e^ due to mainly the presence of unobservable factors in the infant health equation that are correlated with the adequacy of prenatal care use chosen by the mother [31]. If this is indeed the case, the estimated regression coefficients in our model will be inconsistent, and we can also not infer causality between the dependent variable and the independent variables [32]. Since controlling for endogeneity matters in empirical studies [26], we employ the Two–Stage–Residual–Inclusion (2SRI) method [33] in an attempt to correct for this endogeneity. For simplicity, we assume that this is the only endogenous covariate in our model. In the 2SRI method, we control for potential endogeneity of prenatal care use by computing the generalized residuals^f^ from the adequacy of prenatal care model and including these generalized residuals as an additional regressor in the birth weight model.

Following [34], we test for the endogeneity of the adequacy of prenatal care use in the birth weight equation by testing for the statistical significance of these residuals in the equation. If the coefficient of the residuals is statistically significantly different from zero, then the adequacy of prenatal care use variable is endogenous; otherwise, it is exogenous.

#### Unobserved heterogeneity

In our case, unobserved heterogeneity will exist if there are some unobservable factors that interact non–linearly with the adequacy of prenatal care use causing the effect of prenatal care use on birth weight to differ amongst children in the population [27].

The standard procedure for controlling for unobserved heterogeneity is the control function approach^g^ [3,35]. We employ this approach.

### Model identification

For us to properly interpret the estimated parameters of our birth weight model, it is important that birth weight effects of the endogenous covariate (in our case, the adequacy of prenatal care use) and of the sample selection rule be identified [3]. Because we have one endogenous variable in our model, identification requires at least two exclusion restrictions since we have a situation that requires the simultaneous solution of two equations [3].

The variables chosen as instruments should be uncorrelated with the stochastic error term in the birth weight equation (i.e. they should be valid or exogenous), should be correlated with the endogenous variable in the birth weight equation (i.e. they should be relevant, or rather, their effects on the endogenous explanatory variable in the birth weight equation should be statistically significant), and should be excluded from the birth weight equation [3,25,36,37].

In our case, therefore, the variables we use as instruments for prenatal care use should first, affect prenatal care use or be associated with prenatal care use; second, they should be unrelated to mother or household characteristics; and third, they should be related to birth weight only through their association with prenatal care [37].

Examples of variables that have been used as instruments for prenatal care in the literature include number of prenatal care clinics or providers per capita, distance from residence to prenatal care clinics, population per hospital bed, unemployment rate, rate of uninsured females, price of prenatal care, bus strikes, whether mother cohabits with father of child, and mother’s income [17,21,38].

We use the “average distance to the nearest health facility” and the “health facilities per 100,000 of population” as instruments in our models. Our models are, therefore, exactly identified [36]. We use these instruments both to identify birth weight reporting and also to identify the effect of prenatal care on birth weight.

The choice of distance to the nearest health facility as an instrument is based on the assumption that distances to health facilities are correlated with prenatal care use. Since mothers have other uses for their time (such as engaging in paid work, housework, and child care), they must optimally allocate the time available to them amongst the various uses. The longer the distance to the nearest health facility, the higher the opportunity cost to the mother of visiting the facility for prenatal care. Research actually shows that distance to the health facility significantly influences the utilization of health care services (see, for example, [39]). We would, therefore, expect a mother’s utilization of prenatal care to be limited the longer the distance to the nearest health facility. Consequently, we expect a mother’s utilization of prenatal care to be inadequate the longer the distance to the nearest health facility.

One argument in the literature against the use of distance to the nearest health facility as an instrumental variable is that mothers can choose to live near health facilities because of their health status or because of their preferences [15,23]. This then undermines the argument that the distances are exogenous.

To overcome this possibility, we use provincial^h^ level averages for the distance to the nearest health facility in Kenya. This is because, even though an individual mother may choose to live near a health facility because of her health status or simply because she prefers to do so, all the women in a province are unlikely to make this decision simultaneously every time they are pregnant. As such, an individual woman’s decision may not immediately affect the average distance to the nearest health facility in a province. Furthermore, if the relocation of a mother is from one area of the province to another area of the province, this does not change the average distance to the nearest health facility inthe province.

The health facilities per 100,000 of population is aimed at indicating the overall accessibility and availability of health care in a particular province. We expect that the higher the number of health facilities per 100,000 of population, the more the health care (including prenatal care) is accessible and available for use. Consequently, we expect that the higher the number of health facilities per 100,000 of population, the higher the probability of adequate prenatal care use, and the higher the probability of reporting birth weight.

### Empirical model

We formulate both a single–level model and a multilevel model of birth weight.

#### Single–level model

Since we are using birth weight as a measure of the infant’s health status, we let *H*_*i*_ be the birth weight of the *i*^*th*^ infant. Our *single–level* version of Equation (2) is, therefore, given by:

(10) $$ H_{i}=\beta_{1}+\beta_{2}Z_{i}+\beta_{3}Y + \varepsilon_{1i}  $$

where *Z* is an indicator of the adequacy of prenatal care use, *Y* is a vector of other factors (controls), and *ε*_1_ is a stochastic error term.

Because *Z* is potentially endogenous in Equation (10), we have to control for this potential endogeneity. To use the Two–Stage–Residual–Inclusion method to control for this potential endogeneity, we estimate a model for the adequacy of prenatal care use, obtain generalized residuals from the estimated model using the procedure in [40], and then include these generalized residuals together with the adequacy of prenatal care variable in our structural equation of interest.

The adequacy of prenatal care use variable is constructed based on the WHO recommendations [14]. The adequacy of prenatal care variable is defined as follows:

(11) $$  Z_{i}\,=\, \left\{ \begin{array}{ll} \!1 & \text{if mother sought adequate prenatal care while pregnant}, \\ \!0\! & \text{otherwise}. \end{array} \right.  $$

The appropriate model for the adequacy of prenatal care use is, therefore, the binary regression model [41,42].

Three common methods for deriving the binary regression model include assuming that there is an unobserved variable that is linked to the observed outcome through a measurement equation, constructing the model as a probability model, and generating the model as a random utility model [42], p.132. We adopt the latent variable method because of its appeal to intuition.

Using the latent variable formulation, we can define a latent variable $Z^{*}_{i}$ that is related to *Z*_*i*_ via the following equation:

(12) $$ Z_{i}= \left\{ \begin{array}{ll} 1 & \text{if} \,\, Z_{i}^{*}>0, \\ 0 & \text{otherwise}. \end{array} \right.  $$

This latent variable is linked to the covariates using the equation

(13) $$ Z_{i}^{*}=\alpha_{1}+\alpha_{2}Y + \alpha_{2}Q + \varepsilon_{2i}  $$

where *Y* is a vector of controls, *Q* is a vector of instruments, and *ε*_2_ is a stochastic error term.

Assuming a standard normal distribution for *ε*_2_ leads to a probit model given by:

(14) $$ Pr\left(Z_{i}=1\right) =\Phi\left(\alpha_{1}+\alpha_{2}Y + \alpha_{2}Q \right)  $$

We estimate this model, obtain its generalized residuals, and include the generalized residuals as an additional variable in the structural equation of interest.

To control for possible non–random selection of individuals into the estimation sample, we also estimate a sample selection equation. Let selection into the sample be given by the following

(15) $$ {Rbw}_{i}= \left\{ \begin{array}{ll} 1 & \text{if infant \textit{i}'s birth weight is reported},\\ 0 & \text{otherwise}. \end{array} \right.  $$

Following [30], we formulate a linear probability sample selection model as:

(16) $$ {Rbw}_{i}=\gamma_{1}+\gamma_{2}Y + \gamma_{3} Q + \upsilon_{3i}  $$

where *Y* is a vector of controls, *Q* is a vector of instruments, and *υ*_3_ is a stochastic error term.

We estimate this model by Ordinary Least Squares, obtain the predicted probabilities, $\hat {P}$, construct the selection term, $\left (\hat {P}-1\right)$, and include this selection term as an additional regressor in our model of primary interest [30].

To control for potential unobserved heterogeneity, we include the interaction of the adequacy of prenatal care use with the generalized residuals from the adequacy of prenatal care use equation.

Equation (10) is, therefore, extended as follows:

(17) $$ H_{i}=\beta_{1}+\beta_{2}Z_{i}+\beta_{3}Y +\beta_{4}\hat{\varepsilon}_{2i}+\beta_{5}\left(\hat{P}-1\right) +\beta_{6}Z\hat{\varepsilon}_{2i} + \varepsilon_{1i}  $$

where *Z* is an indicator of the adequacy of prenatal care use, *Y* is a vector of controls, $\hat {\varepsilon }_{2}$ are generalized residuals from the prenatal care model, $\left (\hat {P}-1\right)$ is the selection term, and *ε*_1_ is a stochastic error term. When necessary, Equation (17) is extended by the inclusion of additional higher order interaction terms between the adequacy of prenatal care use and the generalized residuals computed from the adequacy of prenatal care use equation.

#### Multi–level model

We obtain the random–intercept multilevel models by breaking the stochastic error terms in our single–level models into two parts, a mother–specific component, *ζ*, and an infant–specific component, *ε*. The mother–specific component, *ζ*, controls for unobservable mother–specific characteristics that affect the dependent variable of interest (e.g. birth weight, adequacy of prenatal care use, reporting of birth weight) and is assumed to remain unchanged across infants born to the same mother but to be independent across mothers [43]. The infant–specific component, *ε*, varies between infants as well as mothers but is assumed to be independent across both infants and mothers [43]. It is also further assumed that *ζ* is independent of *ε* [43].

Letting *H*_*ij*_ be the birth weight of the *i*^*th*^ child born to the *j*^*th*^ mother, the multilevel counterparts of our models are as follows:

(18) $$ H_{ij}=\beta_{1}+\beta_{2}Z_{ij}+\beta_{3}Y+\zeta_{1j}+\epsilon_{1ij}  $$

(19) $$ {\qquad}{\qquad}{\qquad}Z_{ij}\,=\, \left\{ \begin{array}{ll} 1 & \text{if mother \textit{j} sought adequate prenatal care when pregnant with infant \textit{i}},\\ 0 & \text{otherwise}. \end{array} \right.  $$

(20) $$ {\small{{} \begin{aligned} &{Rbw}_{ij}\\ &\;\;= \!\left\{\! \begin{array}{ll} 1 & \text{if the birth weight for infant \textit{i} from mother \textit{j} is reported},\\ 0 & \text{otherwise}. \end{array} \right. \end{aligned}}}  $$

For the multilevel case, the binary responses are related to the latent continuous responses via the following equations:

(21) $$ Z_{ij}= \left\{ \begin{array}{ll} 1 & \text{if}\,\, Z_{ij}^{*}>0,\\ 0 & \text{otherwise}. \end{array} \right.  $$

The multilevel latent response for the adequacy of prenatal care use, and the multilevel sample–selection models are given by:

(22) $$ Z_{ij}^{*}=\alpha_{1}+\alpha_{2}Y + \alpha_{2}Q+\zeta_{2j}+ \epsilon_{2ij}  $$

(23) $$ {Rbw}_{ij}=\gamma_{1}+\gamma_{2}Y + \gamma_{3} Q + \zeta_{3j}+\epsilon_{3ij}  $$

where *Y* is a vector of controls, *Q* is a vector of instruments, *ζ*_1*j*_,*ζ*_2*j*_,*ζ*_3*j*_ are random intercepts that control for unobservable mother – specific characteristics, *ε*_1*ij*_,*ε*_2*ij*_,*ε*_3*ij*_ are infant – specific stochastic error terms.

We assume that *ζ*_1*j*_∼*N*(0,*ψ*_1_), *ζ*_2*j*_∼*N*(0,*ψ*_2_), and *ζ*_3*j*_∼*N*(0,*ψ*_3_). *ε*_1*ij*_∼*N*(0,*θ*), while *ε*_2*ij*_ and *ε*_3*ij*_ are assumed to follow the standard normal distribution.

The corresponding multilevel probit model for the adequacy of prenatal care use is given by:

(24) $$ Pr\left(Z_{ij}=1\right) =\Phi\left(\alpha_{1}+\alpha_{2}Y + \alpha_{3} Q+\zeta_{2j}\right)  $$

To control for potential endogeneity of prenatal care, potential sample selection bias and potential unobserved heterogeneity, we extend Equation (18) as follows:

(25) $$ {\small{\begin{aligned} H_{ij}\,=\,\beta_{1}&+\beta_{2}Z_{ij}+\beta_{3}Y + \beta_{4}\hat{\epsilon}_{2ij}+\beta_{5}Z_{ij}\hat{\epsilon}_{2ij}+\beta_{6}\left(\hat{P}-1\right)\\ \!\!&+\zeta_{1j}+\epsilon_{1ij} \end{aligned}}}  $$

where $ \hat {\epsilon }_{2ij} $ are generalized residuals from the multilevel prenatal care model, and $\left (\hat {P}-1\right)$ is the selection term.

For the multilevel models, the dependence among the responses for the same mother can be quantified by the residual intraclass correlation, *ρ*, of the responses given the covariates [43]. For the multilevel birth weight model, this is given by:

(26) $$ \rho=\frac{\psi}{\psi+\theta}  $$

while for the multilevel binary models it is given by:

(27) $$ \rho=\frac{\psi}{\psi+1}.  $$

We estimate our models using Stata software version 12 [44]. The multilevel binary models are estimated using the gllamm command [43].

### Data

The main dataset we use is the Demographic and Health Survey (DHS) data set for Kenya collected in 2008 [45]^i^. A good guide to Demographic and Health Survey (DHS) data sets can be found in [46]. Demographic and Health Surveys are nationally representative household surveys that provide a wide range of household level data on child and maternal health.

Data on average distance to health facilities is obtained from the community dataset of the Kenya Integrated Household Budget Survey (KIHBS) that was carried out between 2005 and 2006^j^ [47]. Data on health facilities per 100,000 of population is computed using information obtained from the Kenya National Bureau of Statistics [48,49].

Following [14], prenatal care use is classified as “adequate” if all of the following conditions were met: the mother must have sought the prenatal care from a skilled provider, in particular, from either a doctor or a nurse; the mother must have had at least four prenatal care visits; and the first prenatal care visit must have occurred within the first four months of pregnancy.

Table 2 shows the variable definitions for the various variables found in our models.

**Table 2:** **Variable definitions**

**Variable**	**Definition**
Birth weight	Birth weight in grams.
Birth weight reported	1 if child’s birth weight is reported;
	0 otherwise.
Adequate prenatal care	1 if prenatal care is sought from a skilled
	provider (doctor or nurse), the total
	number of visits is at least four, and the
	first prenatal care visit occurs within four
	months of pregnancy; 0 otherwise.
Mother’s age at birth	Mother’s age at time of birth of child in
of child	years.
Birth order	Child’s birth order.
Twin or multiple birth	1 if child is twin or from a multiple birth,
child	0 otherwise.
Urban residence	1 if area of residence is urban; 0 otherwise.
No education	1 if mother has no formal schooling;
	0 otherwise.
Primary education	1 if mother’s highest education level is
	primary; 0 otherwise.
Secondary education	1 if mother’s highest education level is
	secondary; 0 otherwise.
Higher education	1 if mother’s highest education level is
	higher; 0 otherwise.
Number of living	Number of living children born to mother.
children	
Never married	1 if mother has never been married;
	0 otherwise.
Final say on own	1 if mother has final say on own health
health care	care; 0 otherwise.
First born child	1 if child is first born; 0 otherwise.
Male child	1 if sex of child is male; 0 otherwise.
Preceding birth interval	The interval in months between birth of
	the child under study and the immediate
	preceding birth to the mother.
Mother wanted	1 if Yes; 0 otherwise.
pregnancy	
Wealth index	Household’s wealth index, ranges from 1
	to 5.
Average distance to	Provincial level average distance to
nearest health facility	nearest health facility in kilometres.
Health facilities per	Number of health facilities per 100,000 of
100,000 of population	population, measured at the provincial
	level.
Selection term	Term constructed from the selection
	model that controls for sample selection
	bias.
Prenatal care residual	Generalized residuals from the prenatal
	care model.

### Estimation strategy

We estimate our models in two stages. In the first stage, we estimate sample selection models and prenatal care models. In the second stage, we estimate the birth weight model.

## Results

In this section we present the descriptive statistics, the results of the first–stage models, and the results of the birth weight model.

### Descriptive statistics

The descriptive statistics are shown in Table 3.

**Table 3:** **Descriptive statistics**

**Variable**	**Number of**	**Mean**	**Standard**	**Minimum**	**Maximum**
	**observations**		**deviation**		
Birth weight	2,741	3,320.245	682.365	850	8000
Birth weight reported	5,706	0.480	0.500	0	1
Adequate prenatal care	5,706	0.169	0.375	0	1
Mother’s age at birth of child	5,706	26.245	6.496	12	48
Urban residence	5,706	0.243	0.429	0	1
No education	5,706	0.214	0.41	0	1
Primary education	5,706	0.562	0.496	0	1
Secondary education	5,706	0.169	0.375	0	1
Higher education	5,706	0.055	0.228	0	1
Final say on own health care	5,706	0.211	0.408	0	1
First born child	5,706	0.230	0.421	0	1
Male child	5,706	0.512	0.500	0	1
Wealth index	5,706	2.817	1.518	1	5
Average distance to nearest health facility	5,706	8.709	5.381	3.11	22.64
Health facilities per 100,000 of population	5,706	13.225	2.895	8	21

From the table, we can observe that the average birth weight in the sample is 3,320 grams. We can further observe that about 48% of the children had their birth weights reported while about 16.9% of the infants were born to mothers who had sought adequate prenatal care when pregnant. The table also shows that the average age at birth for mothers is about 26 years and about 51% of the infants in the sample are males.

### First–stage models

We report the average marginal effects^k^ based on our estimations [41].

Table 4 shows the estimation results for the sample selection model and the adequacy of prenatal care model for our sample.

**Table 4:** **Average marginal effects for sample selection and prenatal care models, robust** ***Z*** **statistics in parentheses**

**Variable**	**Report**		**Adequate**	
	**(birth weight = 1)**		**(prenatal care = 1)**	
	**(1)**	**(2)**	**(3)**	**(4)**
Mother’s age at birth of child	0.005	0.009	0.018	0.018
	(0.75)	(1.19)	(2.80)	(2.78)
Square of mother’s age at birth of child	-0.00007	-0.0001	-0.0003	-0.0003
	(-0.57)	(-0.97)	(-2.50)	(-2.50)
Urban residence	0.079	0.064	-0.014	-0.014
	(4.17)	(3.07)	(-0.89)	(-0.90)
Primary education	0.142	0.14	0.040	0.040
	(8.05)	(6.64)	(2.47)	(2.46)
Secondary education	0.319	0.306	0.092	0.092
	(14.04)	(11.70)	(4.83)	(4.76)
Higher education	0.386	0.368	0.211	0.211
	(15.34)	(10.24)	(8.75)	(8.67)
Final say on own health care	0.0005	-0.007	0.001	0.001
	(0.03)	(-0.45)	(0.09)	(0.09)
First born child	0.099	0.088	0.006	0.006
	(5.92)	(5.84)	(0.45)	(0.46)
Male child	0.011	0.003	0.004	0.004
	(0.97)	(0.34)	(0.38)	(0.38)
Wealth index	0.084	0.092	0.021	0.021
	(14.02)	(14.23)	(4.41)	(4.45)
Average distance to nearest health facility	-0.024	-0.022	-0.011	-0.011
	(-5.09)	(-4.15)	(-2.78)	(-2.75)
Average distance to nearest health facility squared	0.001	0.0009	0.0003	0.0003
	(5.52)	(4.45)	(1.74)	(1.73)
Health facilities per 100,000 of population	0.083	0.070	0.073	0.073
	(4.98)	(4.43)	(5.30)	(5.33)
Health facilities per 100,000 of population squared	-0.002	-0.002	-0.002	-0.002
	(-3.81)	(-3.43)	(-5.08)	(-5.11)
*ψ*		0.109		2.084×10^−20^
*ρ*		0.59		2.084×10^−20^
**Likelihood ratio test for** ***ρ*** ***=0*** **:** $ \boldsymbol {{\chi ^{2}_{1}}}$		788.19		4.5×10^−4^
(P-value)		(0.00)		(0.492)
Number of observations	5,706	5,706	5,706	5,706

The estimates for the sample selection model come from a linear probability model while those of the prenatal care model come from a probit model.

We show the results for the multilevel model and those for the single level model, for comparison purposes. The single level model results are shown in columns (1) and (3) of the table while the multilevel model results are shown in columns (2) and (4) of the table.

We show the results for the sample selection model in Columns (1) and (2) of the table and those of the prenatal care model in columns (3) and (4) of the table. From columns (1) and (2) we can conclude that mothers who have formal education, reside in urban, or are members of wealthy households are more likely to report the infant’s birth weight, holding other factors constant. The birth weight of a first born child is also more likely to be reported than that of a non–first born child, holding other factors constant.

Columns (3) and (4) show that significant determinants of adequate prenatal care use include mother’s age at birth of child, level of education, wealth index, average distance to nearest health facility, and health facilities per 100,000 of population.

The likelihood ratio test for *ρ*=0 shown in the table is a test of the null hypothesis that the variance of the random intercept is zero. From the table, we can observe that while this hypothesis is rejected in the sample selection model, we are unable to reject it in the prenatal care model.

### Birth weight model

Table 5 shows the results for the single–level birth weight model.

**Table 5:** **Average marginal effects from single–level birth weight model, robust** ***Z*** **statistics in parentheses**

**Variable**	**Birth weight (grams)**				
	**(1)**	**(2)**	**(3)**	**(4)**	**(5)**
Adequate prenatal care	0.304	10.854	414.087	475.45	2205.127
	(0.01)	(0.37)	(1.49)	(1.54)	(2.18)
Mother’s age at birth of child	-45.88	-22.297	-29.058	-31.103	-31.156
	(-2.49)	(-1.21)	(-1.51)	(-1.56)	(-1.56)
Square of mother’s age at birth of child	0.724	0.402	0.505	0.536	0.535
	(2.29)	(1.28)	(1.55)	(1.60)	(1.60)
Urban residence	-61.365	106.096	111.614	114.174	114.346
	(-1.80)	(2.77)	(2.93)	(2.99)	(2.99)
Primary education	141.552	361.303	358.544	357.8	355.392
	(2.96)	(6.76)	(6.69)	(6.66)	(6.51)
Secondary education	124.549	651.331	644.921	643.041	648.9
	(2.33)	(8.18)	(8.04)	(7.97)	(7.97)
Higher education	76.956	731.185	653.588	638.452	612.633
	(1.29)	(7.88)	(5.86)	(5.43)	(5.17)
Final say on own health care	5.415	12.612	11.388	11.086	9.619
	(0.17)	(0.41)	(0.37)	(0.36)	(0.31)
First born child	-91.969	112.88	122.041	124.537	126.843
	(-2.57)	(2.60)	(2.82)	(2.89)	(2.94)
Male child	113.972	134.174	134.26	134.088	135.188
	(4.41)	(5.21)	(5.21)	(5.20)	(5.24)
Wealth index	-12.379	160.905	163.849	164.254	166.391
	(-0.92)	(6.97)	(7.09)	(7.12)	(7.15)
Selection residual		-1882.118	-2016.37	-2050.817	-2071.41
		(-9.00)	(-9.04)	(-8.95)	(-8.87)
Prenatal care residual			-230.673	-314.384	-352.295
			(-1.44)	(-1.32)	(-1.43)
Interaction of prenatal care with residual				63.038	-3991.354
				(0.48)	(-1.67)
Square of Interaction of prenatal care with residual					3075.525
					(1.63)
Cube of Interaction of prenatal care with residual					-736.606
					(-1.54)
Number of observations	2,741	2,741	2,741	2,741	2,741

In the table, the columns showing the results have been labelled (1), (2), (3), (4) and 5. They all show different versions of the model. Column (1) of the table shows the basic model; column (2) shows the version of the model controlling for sample selection bias; column (3) shows the version of the model controlling for both sample selection bias and endogeneity of prenatal care use; column (4) shows the version of the model controlling for sample selection bias, endogeneity of prenatal care use and unobserved heterogeneity; while column (5) shows a version of the model that contains the same variables as the version of the model in column (4) together with higher order terms for controlling for unobserved heterogeneity.

Looking at the version of the model in column (2) in the table, we notice that the selection residual is statistically significant at the 5% level of significance implying that the version of the model in column (1) does suffer from selection bias. From the version of the model in column (3), we can conclude that prenatal care is not an endogenous determinant of birth weight since the coefficient of the prenatal care residual is not statistically significant. Looking at the version of the model in column (4) we can conclude that there is no unobserved heterogeneity in our model since the coefficient of the interaction of prenatal care with its residual is not statistically significant. The version of the model in column (5) includes higher order terms for controlling for unobserved heterogeneity. Even though these additional terms are not individually statistically significant, we notice that as a result of inclusion of these terms, prenatal care is now statistically significant. Among all the versions of the model, we choose the version of the model in column (5) as the most appropriate.

Table 6 shows the results for the multi–level birth weight model.

**Table 6:** **Average marginal effects from multi–level birth weight models,** ***Z*** **statistics in parentheses**

**Variable**	**Birth weight (grams)**				
	**(1)**	**(2)**	**(3)**	**(4)**	**(5)**
Adequate prenatal care	-14.36	0.708	301.076	365.457	2121.26
	(-0.48)	(0.02)	(1.04)	(1.11)	(1.72)
Mother’s age at birth of child	-45.413	-13.375	-17.706	-19.669	-19.635
	(-2.59)	(-0.76)	(-0.98)	(-1.05)	(-1.04)
Square of mother’s age at birth of child	0.716	0.264	0.33	0.36	0.358
	(2.38)	(0.88)	(1.08)	(1.14)	(1.13)
Urban residence	-60.869	94.656	98.302	100.768	101.094
	(-1.62)	(2.31)	(2.39)	(2.42)	(2.43)
Primary education	130.635	369.865	369.038	368.947	366.505
	(2.63)	(6.60)	(6.59)	(6.58)	(6.45)
Secondary education	113.868	680.26	678.267	677.87	684.583
	(2.04)	(7.94)	(7.91)	(7.91)	(7.92)
Higher education	81.324	775.407	720.137	706.745	678.97
	(1.19)	(7.37)	(6.11)	(5.78)	(5.38)
Final say on own health care	10.707	0.224	-1.544	-2.146	-3.928
	(0.31)	(0.01)	(-0.05)	(-0.06)	(-0.12)
First born child	-83.587	113.293	120.996	123.401	126.334
	(-2.48)	(2.79)	(2.93)	(2.96)	(3.02)
Male child	117.638	126.283	125.46	124.948	125.745
	(4.78)	(5.18)	(5.14)	(5.11)	(5.14)
Wealth index	-14.082	193.084	197.401	198.632	201.448
	(-1.02)	(6.97)	(7.05)	(7.05)	(7.07)
Selection residual		-2106.914	-2223.24	-2264.1	-2292.424
		(-8.56)	(-8.23)	(-7.85)	(-7.79)
Prenatal care residual			-171.664	-254.642	-300.713
			(-1.04)	(-0.97)	(-1.09)
Interaction of prenatal care with residual				59.945	-4037.807
				(0.41)	(-1.42)
Square of Interaction of prenatal care with residual					3114.758
					(1.40)
Cube of Interaction of prenatal care with residual					-746.803
					(-1.34)
*ψ*	203813.423	182023.396	181393.365	181389.958	181478.556
*ρ*	0.449	0.414	0.413	0.413	0.414
**LR test for** ***ρ*** **=0**: $ {\chi ^{2}_{1}} (P-value) $	133.77 (0.00)	115.08 (0.00)	114.18 (0.00)	114.17 (0.00)	114.43 (0.00)
Number of observations	2,741	2,741	2,741	2,741	2,741

The column of results are also labelled as (1), (2), (3), (4) and (5). Column (1) of the table shows the basic model; column (2) shows the version of the model that controls for sample selection bias; column (3) shows the version of the model that controls for sample selection bias and endogeneity of prenatal care use; while column (4) shows the version of the model that controls for sample selection bias, endogeneity of prenatal care use and unobserved heterogeneity. We include higher order terms that control for unobserved heterogeneity in the version of the model in column (5).

The version of the model in column (5) is the best amongst our models. The results of the likelihood ratio test for *ρ*=0 in the model imply that the multi–level model is appropriate for our analysis. We can observe from the model that although we have a selection issue, prenatal care is not endogenous and our models do not suffer from unobserved heterogeneity. We can, however, observe from the model in column (5) that adequate use of prenatal care increases birth weight.

We show the results of both the single–level birth weight model and the multi–level birth weight model in Table 7, for comparison purposes.

**Table 7:** **Average marginal effects from our chosen birth weight models,** ***Z*** **statistics in parentheses**

**Variable**	**Birth weight (grams)**	
	**Single level**	**Multi–level**
	**model**	**model**
Adequate prenatal care	2205.127	2121.26
	(2.18)	(1.72)
Mother’s age at birth of child	-31.156	-19.635
	(-1.56)	(-1.04)
Square of mother’s age at birth	0.535	0.358
of child	(1.60)	(1.13)
Urban residence	114.346	101.094
	(2.99)	(2.43)
Primary education	355.392	366.505
	(6.51)	(6.45)
Secondary education	648.9	684.583
	(7.97)	(7.92)
Higher education	612.633	678.97
	(5.17)	(5.38)
Final say on own health care	9.619	-3.928
	(0.31)	(-0.12)
First born child	126.843	126.334
	(2.94)	(3.02)
Male child	135.188	125.745
	(5.24)	(5.14)
Wealth index	166.391	201.448
	(7.15)	(7.07)
Selection residual	-2071.41	-2292.424
	(-8.87)	(-7.79)
Prenatal care residual	-352.295	-300.713
	(-1.43)	(-1.09)
Interaction of prenatal care with	-3991.354	-4037.807
residual	(-1.67)	(-1.42)
Square of Interaction of prenatal	3075.525	3114.758
care with residual	(1.63)	(1.40)
Cube of Interaction of prenatal	-736.606	-746.803
care with residual	(-1.54)	(-1.34)
*ψ*		181478.556
*ρ*		0.414
**LR test for** ***ρ*** **=0**: $ {\chi ^{2}_{1}} (P-value) $		114.43 (0.00)
Number of observations	2,741	2,741

From Table 7, we can conclude that significant determinants of birth weight include adequate prenatal care use, urban residence, education, whether or not the child is firstborn, sex of the child, and wealth.

## Discussion

### First–stage models

The results in Table 4 show that the older the mother at the time of birth of the child, the higher the probability of seeking adequate prenatal care, holding other factors constant. This is likely to be mainly because older women are more experienced in matters of child birth and may have learnt from earlier experiences the advantages of seeking adequate prenatal care while pregnant. This finding is supported by the finding in the literature where maternal age of less than 18 years is found to be associated with inadequate use of prenatal care in Aracaju, Northeast Brazil [50].

The results also show that compared to mothers without formal schooling, those with either primary education, secondary education, or higher education, have a higher probability of seeking adequate prenatal care, holding other factors constant. The reason could be that education enables the mothers to be aware of the benefits of prenatal care by, for instance, being able to benefit from awareness campaigns. Findings from the literature support the positive effects of education on the probability of seeking adequate prenatal care. For example, in Aracaju, Northeast Brazil, low maternal schooling is associated with inadequate prenatal care use [50]. Similarly, in Turkey, it is observed that the probability of women with one to five years of schooling and that of the women with six or more years of schooling using prenatal care services is higher than that of the women with no schooling [51].

The results also show that the wealthier the household to which a mother belongs, the higher the probability of seeking adequate prenatal care, holding all other factors constant. This is similar to the finding in the literature that household wealth is positively associated with prenatal care use [51]. The explanation here is that wealthy households have the necessary resources to pay for the indirect costs of using prenatal care services.

The results of the prenatal care model in Table 4 further show that, holding other factors constant, the longer the average distance to the nearest health facility, the lower the probability of the mother seeking adequate prenatal care. This is in line with our expectations. A more likely explanation of this relationship is that the total cost of seeking prenatal care from a facility is higher if the facility is farther from the mother. This is true of the indirect costs such as the cost of transportation to the facility, and of the opportunity cost since it might take longer for the mother to go to such facilities. The findings from the literature support this. For example, [50] reports that those women who had to obtain prenatal care outside Aracaju had inadequate use of prenatal care services.

We can further observe from the results in Table 4 that more health facilities per 100,000 of population increase the probability of seeking adequate prenatal care, if other factors are held constant. This is because more health facilities mean that health care (including prenatal care) is generally available for those who may want to seek it.

### Birth weight model

The results in Table 7 show that adequate use of prenatal care increases birth weight, holding other factors constant. This finding is consistent with the findings in the literature. For example, in Uruguay, [20] find birth weight to be positively related to prenatal care use. It is further shown in the literature that prenatal care increases birth weight in normal pregnancies [38]. The finding implies that prenatal care is only useful to infant health if obtained adequately. Recall that by adequate care we mean that the care is obtained from a skilled provider, the mother makes at least four visits, and the first visit is initiated within four months of pregnancy. The reason for the positive effect of adequate prenatal care on infant health could be mainly that during prenatal care visits, mothers receive a wide range of advice on what to do so as to improve the health of the foetus. They, further, receive treatment from any illnesses which might have detrimental effects on the health of the foetus.

Comparing the results from the multi–level model and those from the single–level model shows that the single–level model overstates the effect of adequate use of prenatal care on birth weight. In the single–level model, holding other factors constant, the birth weight of infants whose mothers sought adequate prenatal care while pregnant is higher than that of the infants whose mothers did not seek adequate prenatal care by about 2205 grams. This implies that adequate use of prenatal care increases birth weight by about 2205 grams, holding other factors constant. In the multilevel model, however, the corresponding difference in birth weights between infants whose mothers sought adequate prenatal care and those whose mothers did not seek adequate prenatal care is only about 2121 grams. Consequently, failure to control for unobserved mother–specific characteristics, leads to an overstatement of the effect of adequate prenatal care use on birth weight.

The results further indicate that mothers who reside in urban areas have heavier children compared to those who reside in rural areas, holding other factors constant. A possible explanation would be the relative availability of skilled health providers in urban areas than in rural areas leading to prompt treatment of all sorts of illnesses that could be detrimental to child health. There is also the issue of the relative high levels of awareness in urban areas than in rural areas of child health matters due to having so many information campaigns.

The results also indicate that mothers with formal schooling have heavier infants compared to those without formal schooling, holding other factors constant. This result is consistent with some of the findings in the literature where, for example, in Malawi, women who have attained at least secondary level education are less likely to bear low birth weight children compared to women without formal education [52].

The results indicate that male infants have higher birth weights compared to female infants, holding other factors constant. This is in line with the findings from literature where, for example, in Kenya female infants are found to be lighter than male infants [3].

In contrast to the findings in the literature, however, we find that first born infants have higher birth weights than their non–first born counterparts, holding other factors constant. This could be due to the higher (though not statistically significant) probability of seeking adequate prenatal care when pregnant with the first born child reported in Table 4.

## Conclusions

The main conclusion from our study is that using prenatal care adequately when pregnant leads to higher birth weights amongst infants, and by extension, to better infant health. The study, therefore, demonstrates that prenatal care is effective in improving birth weight when used adequately. We can also conclude that there is need for controlling for unobserved mother–specific effects in models that attempt to investigate the effect of prenatal care on birth weight. There is also further need to control for sample selection bias and unobserved heterogeneity in such models.

Because the study shows that adequate use of prenatal care increases birth weight and, by extension, improves infant health, the implication is that policies for promoting adequate use of prenatal care should be pursued. These policies range from ensuring availability of skilled health care providers such as doctors and nurses at prenatal care clinics, reducing the average distances mothers have to cover when seeking prenatal care services, intensifying education of females as a way of empowering women to be able to make the right choices regarding when to seek prenatal care and from whom, and increasing income opportunities for households.

The study provides important lessons for developing countries in the sense that emphasis should be on adequate prenatal care use, and not just prenatal care use. A clear criteria for judging the adequacy of prenatal care use is also provided. 

## Endnotes

^a^ This is the health of children aged one year and below.

^b^ These are factors whose possession or presence is associated with an increased probability of giving birth to a low birth weight infant [10].

^c^ The Olsen approach involves estimation of a linear probability model of the selection equation, obtaining the probability of selection into the sample $\hat {P}$, construction of the selection term $\left (\hat {P}-1\right)$, and inclusion of this selection term as an additional regressor in the infant health equation [30]. A statistically significant coefficient of the selection term indicates sample selection bias.

^d^ Maximum likelihood estimation is biased in small samples and relies on numerical methods which could lead, in some circumstances, to nonconvergence or convergence with a wrong solution [41]. For a further critique of the Heckman procedure, see [53].

^e^ Common causes of endogeneity include failure to include confounder variables in the model, one or more of the explanatory variables being caused by the current dependent variable, and the explanatory variables being measured with error [32].

^f^ Residuals can generally be viewed as being functionally related to the observed values of the dependent variable and the estimated values of the parameters [54]. For models estimated using maximum likelihood (such as probit), deviance–based definitions of residuals are recommended [55]. A detailed discussion on how to compute these residuals for various non–linear models is provided in [40]. Specifically for the probit model, the discussion in [40] implies that for a binary dependent variable *y*, the *i*^*th*^ residual $\hat {u}_{i}$ can be computed as follows

$$\hat{u}_{i}= \left\{ \begin{array}{ll} \frac{\phi\left(x\hat{\beta}\right)}{\Phi\left(x\hat{\beta}\right)} \;\text{if}\; y_{i}=1 \\ \frac{-\phi\left(x\hat{\beta}\right)}{1-\Phi\left(x\hat{\beta}\right)} \;\text{if}\; y_{i}=0 \end{array} \right. $$

where *ϕ* is the probability density function of the standard normal distribution and *Φ* is the cumulative density function of the standard normal distribution.

^g^ The approach involves including in the birth weight equation interactions between the residuals and the endogenous explanatory variable (in our case, the adequacy of prenatal care use). If the coefficient of the resulting interaction term is statistically significantly different from zero, there is unobserved heterogeneity in our birth weight model. If the coefficient is not statistically significantly different from zero, there is no unobserved heterogeneity in our birth weight model.

^h^ The new constitution enacted in Kenya in 2010 abolished provinces.

^i^ More information on Demographic and Health Surveys can be obtained by visiting http://www.measuredhs.com/What-We-Do/Survey-Types/DHS.cfm

^j^ Since prenatal care is sought during pregnancy, the ideal case would have been to obtain data on distances for the year in which the mother was pregnant with the child. A look at the DHS 2008 data shows that the children in the dataset were aged between less than one year and four years. This puts their years of birth to between 2004 and 2008. This would imply the years at which the mothers were pregnant with the children range roughly from 2003 to 2007. The data on distance to the facilities gathered between 2005 and 2006 gives us a rough idea about the ease or otherwise of access to health care over the five–year period 2005-2010, since we do not expect massive changes in the distances over the five–year period. This period coincides with the period mothers are likely to have been pregnant with about 63% of the children in our estimation sample. We, therefore, believe that the distance information from the KIHBS 2005/2006 provides a good estimate of the indirect cost of accessing the facilities when the mothers were pregnant for the majority of the children.

^k^ One important question we may want to answer after the estimation of our models is how changes in the explanatory variables affect the probabilities of a positive outcome. This question can be answered by reporting the marginal effects of the respective covariates [41]. The marginal effect is computed by taking the partial derivative of the dependent variable or in the case of the binary regression model, taking the partial derivative of the estimated probability model, with respect to the variable of interest [41]. Since in the case of the binary regression model the resulting partial derivative is a function of all the variables, it can either be evaluated at the means of the various variables, leading to what is called the marginal effect at the means, or it can be computed for each observation and then averaged over all observations, leading to average marginal effects [41]. The average marginal effects are preferable to the marginal effects at means [56]. We, therefore, compute and report the average marginal effects for the variables in our models. In the linear regression model, the marginal effects are generally equivalent to the estimated partial slope parameters. For dummy explanatory variables in the binary regression model, the marginal effects are given by the differences in the probabilities when the variable assumes the value of 1 and when it assumes the value of 0 [41].
